# AI-based automated bleeding monitoring in conventional and robot-assisted laparoscopic surgery: a systematic review

**DOI:** 10.1007/s11701-026-03469-4

**Published:** 2026-05-23

**Authors:** Prosper Magara, Sudesh Sivarasu, Lamees Ras, Ahmed Biyabani, Abby Blocker, Majahonkhe Shabangu, Bessie Malila

**Affiliations:** 1 Biomedical Engineering Research Centre, Division of Biomedical Engineering, Department of Human Biology, Faculty of Health Sciences, University of Cape Town, Observatory, Cape Town, 7925 Western Cape South Africa; 2 College of Engineering, Carnegie Mellon University Africa, Regional ICT Center of Excellence Bldg, Kigali Innovation City, Kigali, Rwanda; 3 Department of Electrical and Engineering Sciences, University of Johannesburg, Auckland Park Kingsway Campus, Auckland Park, Johannesburg, Gauteng South Africa; 4 International Centre for Genetic Engineering and Biotechnology, Observatory, Cape Town, 7925 Western Cape South Africa; 5 Department of Obstetrics and Gynaecology, University of Cape Town, Cape Town, South Africa

**Keywords:** Artificial intelligence, Conventional and robotic-assisted laparoscopic surgery, Intraoperative bleeding, Minimally invasive surgery, Estimated blood loss

## Abstract

Artificial intelligence has emerged as a promising approach for improving the detection and management of intraoperative bleeding during conventional and robotic-assisted laparoscopic surgery, where delayed recognition of hemorrhage can lead to increased morbidity and procedural complexity. This review synthesizes current evidence on the use of artificial intelligence for intraoperative bleeding monitoring, with a particular focus on performance, feasibility, and clinical integration. A systematic review was conducted in accordance with PRISMA 2020 guidelines. Comprehensive searches of PubMed, Scopus, Web of Science, IEEE Xplore, Embase, and grey literature identified studies published between 2016 and 2025 that applied artificial intelligence to bleeding prediction, detection, localization, tracking, and quantitative blood-loss estimation during conventional and robotic-assisted laparoscopic surgery. Data relating to study design, model architectures, evaluation metrics, latency, and integration feasibility were extracted and summarized narratively. Across the included studies, artificial intelligence models demonstrated high detection accuracy in predominantly single-centre, retrospective, or simulation-based settings, with several approaches reporting promising real-time feasibility under controlled experimental conditions. Emerging work also explored bleeding source tracking, blood-loss estimation, and early integration into surgical workflows. However, most studies relied on small, single-center datasets and retrospective validation, limiting generalizability and clinical translation. Overall, artificial intelligence–based bleeding monitoring in conventional and robotic-assisted laparoscopic surgery shows increasing technical maturity and potential, though largely unvalidated in prospective clinical settings. Future research should prioritize large, multi-institutional datasets, prospective clinical evaluation, and optimized low-latency deployment within real surgical workflows to support safe and effective intraoperative use.

**Supplementary Information:**

The online version contains supplementary material available at 10.1007/s11701-026-03469-4.

## Introduction

Laparoscopic surgery is a minimally invasive surgical technique in which surgical operations are performed through small incisions on the abdomen using a laparoscopic camera and specialized instruments [1]. The laparoscopic camera provides a real-time view of the patient’s internal organs on a monitor, which allows the surgeon to operate with precision. This technique typically leads to smaller scars, faster recovery times, and fewer postoperative complications compared to traditional open surgery [2]. Laparoscopic surgery can either be robotic-assisted or performed without robotic assistance [3]. However, laparoscopic surgery requires a high level of expertise due to its steep learning curve, which includes mastering hand-eye coordination, operating in constrained anatomical spaces, and using specialized tools while viewing a two-dimensional (2D) video feed [4]. One limitation to the widespread adoption of laparoscopic surgery is concern over uncontrollable intraoperative bleeding events in procedures such as laparoscopic hepato-pancreato-biliary surgery [5], laparoscopic cholecystectomy, and laparoscopic rectal resection [2]. This risk of uncontrollable bleeding, particularly when the procedure is conducted by a novice surgeon, raises patient safety concerns [6]. Evidence suggests that intraoperative bleeding accounts for 23% of adverse events in laparoscopic surgery [7].

Bleeding during laparoscopic surgery is a significant issue because it can be difficult to detect and control due to the limited field of view and constrained working space [6]. Unlike open surgery, the small incisions used in this minimally invasive technique reduce the surgeon’s ability to directly visualize the bleeding site [6]. In addition, the formation of pneumoperitoneum with carbon dioxide, which is necessary for laparoscopic surgery, can temporarily mask smaller bleeds due to pressure exerted on the bleeding site; these bleeds can become clinically significant once the pressure is released [8]. If not properly managed, intraoperative bleeding can lead to complications, longer surgery times, and conversion to open surgery. Intraoperative bleeding can also cause postoperative complications such as internal bleeding and infection, which compromise patient safety [9] and may necessitate more complex postoperative care and blood transfusion [10].

Conventional and robotic-assisted laparoscopic surgery have evolved significantly with the integration of artificial intelligence (AI) approaches. Automatic bleeding monitoring based on AI in laparoscopic surgery is one such advancement. Despite progress in AI-based bleeding monitoring during conventional and robotic-assisted laparoscopic surgery, timely and precise bleeding detection during laparoscopic procedures remains inadequately addressed. Existing methods often rely on static image analysis [11], which limits real-time applicability [12, 13]. Competing perspectives exist regarding optimal AI architectures and data modalities, with some advocating for multi-task learning models [14], while others emphasize spatiotemporal models that use optical flow for dynamic bleeding point localization [13]. The conceptual framework underpinning this review is the intersection of AI-driven image and video analysis, surgical workflow understanding, and real-time decision support.

The overarching aim of this systematic review was to collect, evaluate, and synthesize existing evidence on AI methods and classical computer vision methods for intraoperative bleeding monitoring during conventional and robotic-assisted laparoscopic surgery. By consolidating findings in various studies, the review provides a comprehensive overview of the latest AI technologies for monitoring bleeding during conventional and robotic-assisted laparoscopic surgery, to support evidence-based decision-making for researchers and clinicians seeking to implement AI solutions for monitoring bleeding in conventional and robotic-assisted laparoscopic surgical workflows.

## Scope of the review

### Aim

This systematic review aims to collect, evaluate and synthesize the current evidence on the use of AI for intraoperative bleeding monitoring during conventional and robotic-assisted laparoscopic surgery.

### Research questions

This systematic review was guided by predefined research questions that structured the literature search, study selection, data extraction, and synthesis. The questions were formulated to capture the range of artificial intelligence tasks applied to intraoperative bleeding monitoring during conventional and robotic-assisted laparoscopic surgery and how the AI based systems perform.

1. What AI-based and computer vision–based systems exist for bleeding monitoring, and which monitoring tasks do they perform?
2. What performance metrics are used to evaluate these systems.
3. Do the AI based systems meet the performance requirements in real-time conventional and robotic-assisted laparoscopic surgery and how do they perform in comparison to classical computer vision methods.

## Methodology of literature selection

This systematic review was conducted in accordance with the Preferred Reporting Items for Systematic Reviews and Meta-Analyses (PRISMA) guidelines [15]. The protocol for this review was registered on the Open Science Framework (OSF) registries with registration number, NCEF8 (https://doi.org/10.17605/OSF.IO/NCEF8) [16, 17]. The completed PRISMA 2020 checklist is provided as Supplementary Material.

### Eligibility criteria

Eligibility was defined using the Population, Intervention, Comparator, and Outcomes (PICO) framework [18]. The population comprised patients, animal models, and simulation-based datasets in which intraoperative bleeding occurred. Robotic-assisted laparoscopic procedures were included within scope. Intervention (I) was defined as the application of AI and classical computer vision (CV) systems to detect, predict, track, and quantify bleeding. Comparators (C) included traditional monitoring approaches, other algorithmic baselines, or no comparator, depending on the study design. The outcomes (O) of interest were model performance metrics, namely accuracy, sensitivity, specificity, F1 score, latency, and clinical feasibility. The primary outcome of interest was AI model detection performance. Secondary outcomes included latency and real-time feasibility, workflow integration feasibility, and dataset characteristics.

Studies were included if they met all of the following: focused on conventional or robotic-assisted laparoscopic surgery; addressed intraoperative bleeding; incorporated AI techniques for bleeding monitoring; used laparoscopic video data or other medical imaging modality data from clinical, animal, or simulated environments; and reported measurable performance outcomes. Publications in English, and peer-reviewed journals and conference proceedings were included. Exclusion criteria included studies outside the laparoscopic surgery domain, absence of AI and classical computer vision methods, editorials, reviews, protocols, non-English articles and non–peer–reviewed articles.

### Search strategy and databases

Literature searches were conducted through PubMed, Scopus, Web of Science, IEEE Xplore, and Embase databases. Additional searches were performed in EBSCOHost, ProQuest, and grey literature sources comprising Google Scholar, arXiv, and MedRxiv. Studies published between January 2016 and 22 July 2025 were eligible for inclusion. The literature search was concluded on 22 July 2025.

The search strategy combined keywords and controlled vocabulary terms relating to laparoscopic surgery, intraoperative bleeding, artificial intelligence, and monitoring tasks. For example, the PubMed search employed the following string: (“laparoscopic surgery” OR “minimally invasive surgery”) AND (“hemorrhage” OR “bleeding” OR “blood loss”) AND (“artificial intelligence” OR “machine learning” OR “deep learning” OR “computer vision”) AND (“detection” OR “prediction” OR “monitoring” OR “quantification” OR “tracking”). Similar Boolean-adapted strategies were applied across other databases. Search results were exported to reference management software, and duplicates were removed prior to screening.

### Screening process

Before the screening process, references retrieved from database searches and grey literature were collected and imported into *Rayyan*, an online systematic review management and screening tool [19]. A total of 332 references were imported. After the references were imported, duplicates of the records retrieved were automatically detected. “Reviewer PM” reviewed duplicates showing more than 50% similarity, and determined if the documents were truly duplicates, after which one of the records was deleted. 117 duplicates were resolved, and 215 records were returned for screening.

The screening process was conducted in two stages. In the first stage, titles and abstracts of all retrieved records were screened against the eligibility criteria. "Reviewer PM and AB" independently assessed each record using *Rayyan*. Records that did not meet the inclusion criteria were excluded, while those considered potentially eligible were advanced to the second stage.

In the second stage, a standardized data extraction form adapted from the Cochrane Handbook for Systematic Reviews of Interventions was developed [20]. Our version was modified to capture details specific to AI studies in laparoscopic surgery, such as input data type, annotation source, AI technique, performance metrics, and validation approach from the remaining records after title and abstract screening. An example of the adapted form is shown in appendix A.The full texts of the remaining studies were retrieved and assessed in detail against the inclusion and exclusion criteria using the standardized data extraction forms. “Reviewer PM” evaluated each article within *Rayyan*, and reasons for exclusion at this stage were documented. “Reviewer AB” assessed the decisions for inclusion or exclusion. Disagreements at both stages were resolved through discussion, with a third reviewer, “Reviewer BM”, who was consulted if consensus could not be reached. Only the studies that met all eligibility requirements after full-text review were included in the final synthesis. Risk of bias and applicability of the included studies were assessed using the Prediction model Risk Of Bias Assessment Tool (PROBAST) framework [21]. Two reviewers PM and AB independently evaluated each study across four domains: Participants, Predictors, Outcome, and Analysis.

**Fig. 1:**
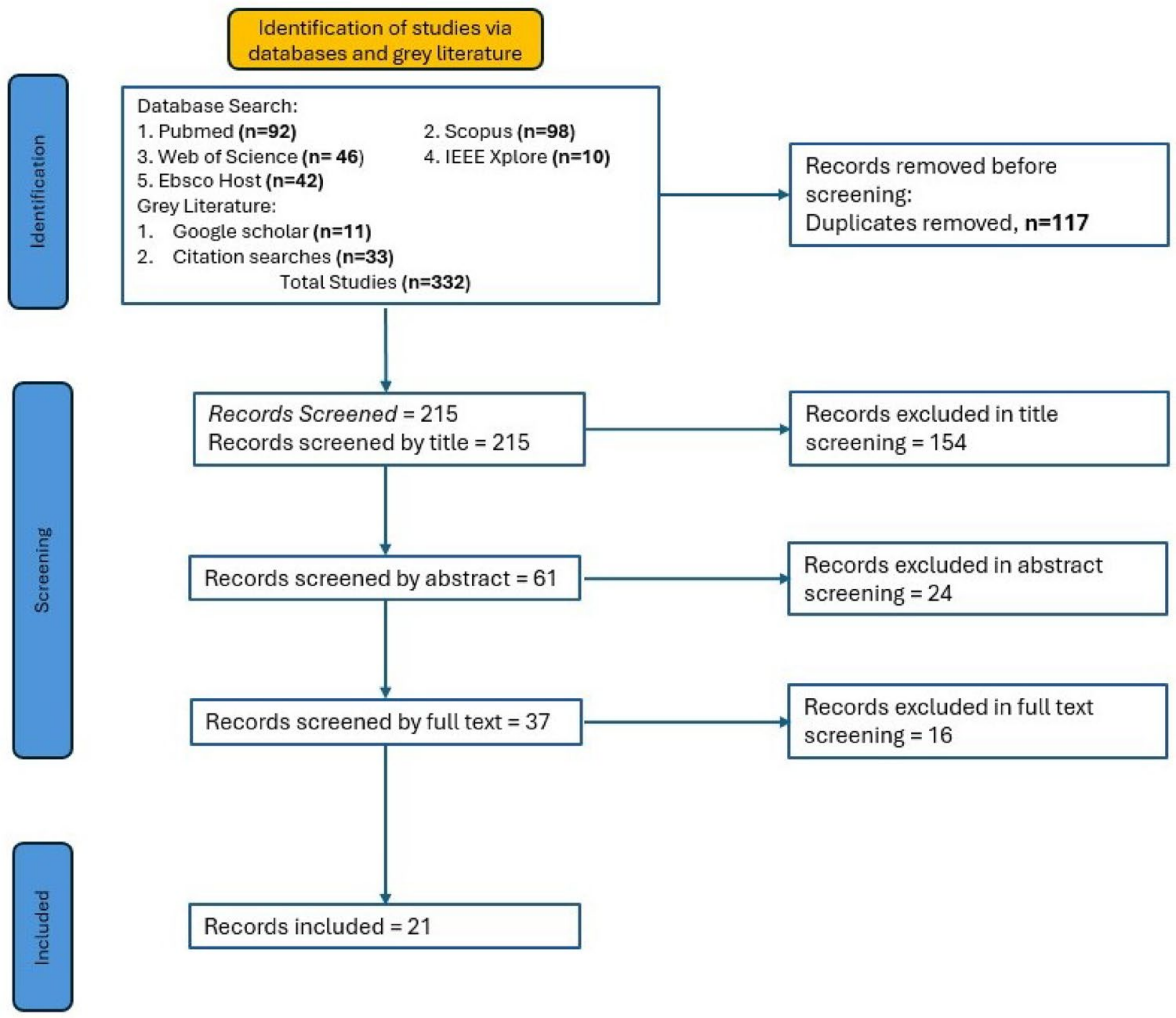
Prisma flow diagram

The screening process was documented in a PRISMA flow diagram shown in Fig. 1. 332 records in total were initially retrieved. 117 duplicates were removed and 215 records were retained. Of the articles screened by title, 154 were removed, leaving 61 records; 24 were excluded based on abstracts, leaving 37 records. Full-text screening was conducted on the 37 remaining records; 16 were excluded, while 21 records were included in this literature review. At this stage, the reasons for exclusion were documented. The reasons for exclusion included that some records focused on general surgery, some focused on endoscopy, and some records used other methods for bleeding monitoring that were not AI-based.

## Results

**Fig. 2:**
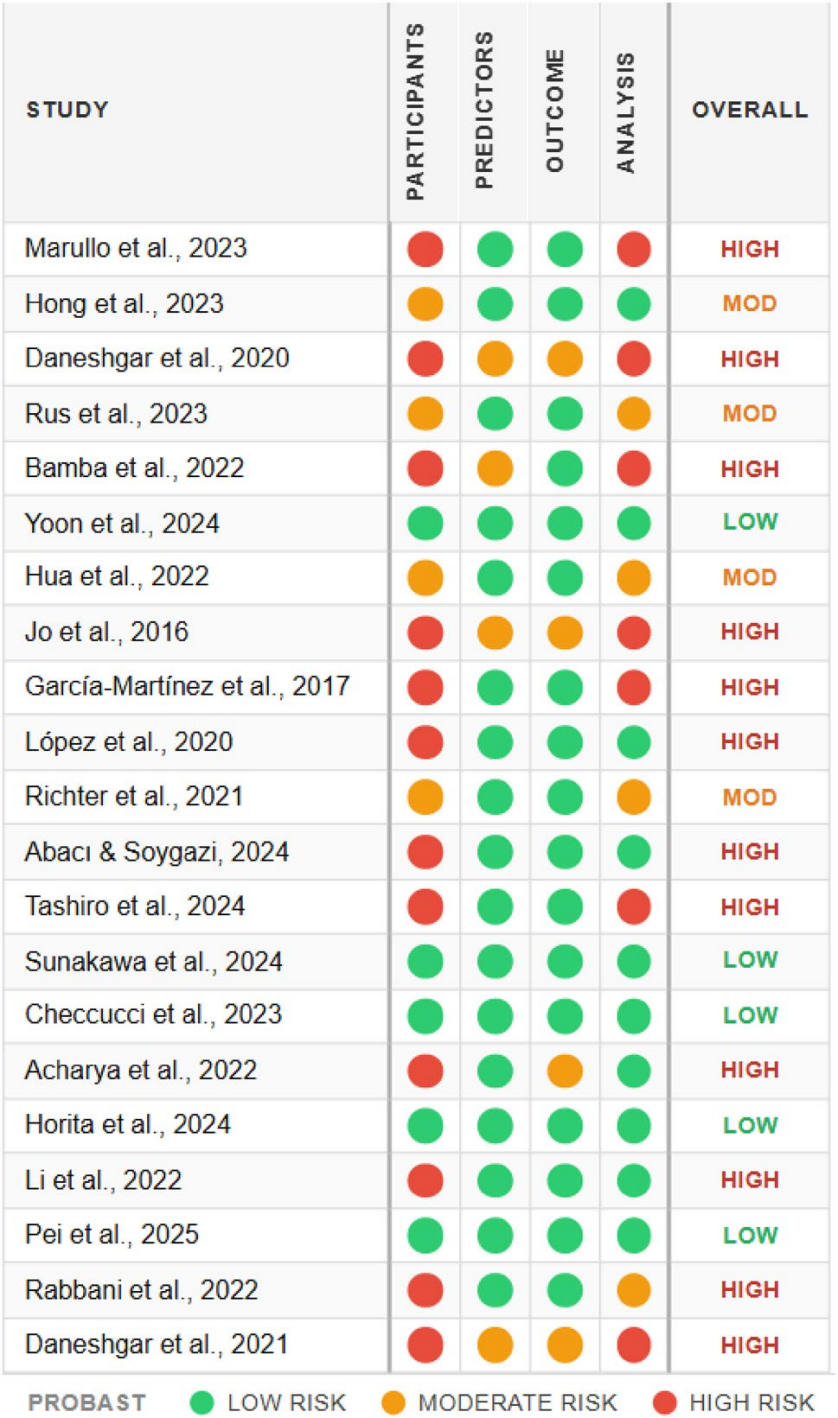
Risk of bias assessment ‘traffic light’ plot for the 21 included articles

The systematic search and screening process identified 21 articles that met the inclusion criteria. Across the 21 included studies, performance varied by task. For bleeding detection, accuracy ranged from 78% to 100%, with F1 scores between 34% and 90% where reported. For blood loss quantification, sensitivity ranged from 96.5% to 98%, specificity from 94% to 98%, and root mean square error (RMSE) from 0.91 to 0.25 g. For source localisation and tracking, Dice scores ranged from 0.53 to 0.77 and IoU from 0.42 to 0.80. Meta-analysis was not feasible due to heterogeneous outcome definitions. Bleeding was variously defined at frame, pixel, and event level, and there was inconsistent reporting of metrics across studies. The study selection workflow is illustrated in the PRISMA flow diagram, Fig. 1. A comprehensive descriptive summary of these studies which details their technical architectures, reported performance, and hardware integration is provided in Table 1. To contextualize the reliability of these findings, the methodological quality and risk of bias for the included literature are summarized in the “traffic light” plot in Fig. 2.

**Table 1:** Descriptive summary of 21 included studies

Study	Detection accuracy	Response time	Integration feasibility	Algorithmic complexity
Marullo et al., 2023 [14]	Dice 81.89%, Acc 90.63%	$\sim$21 FPS (GPU)	Multi-task CNN on surgical videos	Encoder–decoder w/attention (mod)
Hong et al., 2023 [22]	Acc 84.99%, F1 34.04%, Prec 40.18%, Rec 31.29%	NR	Retrospective videos	Fusion model spatio-temporal (high)
Daneshgar et al., 2020 [23]	Acc 97.5%	0.1245 ms	Simulation prototype (Matlab)	Local entropy filter segmentation (low)
Rus et al., 2023 [24]	Prec 0.7293, Rec 0.9641	RT <1s	YOLOv5 on simulator	YOLOv5 object detection
Bamba et al., 2022 [11]	Acc 100%, Prec 98.6%, Rec 87.7%, IoU 96%	RT	IBM Visual Insights/Detectron pipeline	CNN detector/segmenter (mod)
Yoon et al., 2024 [25]	Sens 96.5%, Spec 98.0%, MAE 0.25 g, MAPE 7.26%	NR	Gauze-image workflow	CNN regressor (modified)
Hua et al., 2022 [13]	Prec 0.8373, Rec 0.8034, AP 0.6818, IoU 0.7	RT	Faster R-CNN, video pipeline	Spatio-temporal hybrid model
Jo et al., 2016 [26]	Sens 86%, Spec 59.25%	0.37 s	Matlab prototype	Color segmentation + Otsu (low)
García-Martínez et al., 2017 [27]	Acc 78.26%, Sens 76.47%, Spec 83.33%	24 FPS	OpenCV/ROS; in vivo + in vitro	Pixel color ratios + k-means (low–mod)
López et al., 2020 [28]	Sens >98%, Spec 94%	CNN 4.6 s	Gauze tracking	Classical CV + CNN
Richter et al., 2021 [29]	IoU 78% - 80%	500ms - 800ms	ROS robotic suction	Optical flow + CNN
Abacı & Soygazi, 2024 [30]	RMSE 0.91; R² 0.93	NR	Smartphone-based	Gradient Boosting, RF, SVM, ANN (mod)
Tashiro et al., 2024 [31]	Dice 0.53; IoU 0.42	<0.12 s	LLR overlay	DeepLabv3 segmentation
Sunakawa et al., 2024 [32]	Prec 0.76; Rec 0.79; Dice 0.77	61.7 ms	GPU-based classifier	DeepLabv3+ CNN
Checcucci et al., 2023 [33]	Event Acc 90.63%	NR	Robotic console overlay	Multi-task CNN (mod)
Acharya et al., 2022 [34]	Acc 97.80%; Rec 99.18%; Prec 98.30%	RT	Laparoscopy scenes	Transfer learning (EfficientNet)
Horita et al., 2024 [35]	AP50 0.574	30 FPS	Clinical colectomy videos	YOLOv7 detection
Li et al., 2022 [36]	F1 90%; Sens 90%; PPV 90%	NR	Trocar-mounted device	Tissue classification
Pei et al., 2023 [37]	Dice 64.88%; IoU 78.70%	NR	Region + point dual-branch	BlooDet + optical flow (mod)
Rabbani et al., 2022 [38]	IoU 73.4%; F1 58.09%	NR	Prototype only	STMNet + domain adaptation (mod)
Daneshgar et al., 2021 [39]	Acc 88%; Prec 90%	NR	MATLAB simulation	Local entropy-based

*Abbreviations (performance/metrics):* Acc = accuracy; Prec = precision; Rec = recall; Sens = sensitivity; Spec = specificity; F1 = harmonic mean; AUC = area under ROC curve; Dice = Sørensen–Dice coefficient; IoU = intersection over union; RT = real time; NR = not reported; FPS = frames per second. *Abbreviations (architectures/frameworks):* CNN = convolutional neural network; R-CNN = region-based CNN; YOLOv3/v5/v7 = You Only Look Once; ViT = Vision Transformer; ResNet-50 = Residual Network; DeepLabv3/DeepLabv3+ = segmentation models; GLCM = gray-level co-occurrence matrix; SVM = support vector machine; ROS = Robot Operating System; OpenCV = Computer Vision library; ICG = indocyanine green; LLR = laparoscopic liver resection; STMNet = Space-Time Memory Network

### Bleeding monitoring metrics

#### Prediction

Only three studies focused primarily on bleeding prediction [33, 36, 39]. Checcucci et al. [33] developed a multi-task CNN that can predict the occurrence of bleeding with 90% accuracy. Entropy-based methods achieved reported accuracy of up to 97.5% in simulated environments, while predictive MATLAB prototypes reached 88% accuracy and 90% precision [39]. Li et al. [36] developed a device that utilizes near-infrared spectroscopy to assess hemoglobin parameters at various depths simultaneously and is complemented by a CNN to classify vascular and avascular tissue.

#### Detection

Deep learning approaches achieved high performance, with frame- or event-level accuracy commonly above 90%. Acharya et al. [34], Bamba et al. [11] and Marullo et al. [14] reported accuracy in the 90%−97% range. The Bleeding Artificial Intelligence-based Detector (BLAIR) software achieved detection accuracy of 90.63% [33].

Rus et al. [24] demonstrated recall of 96% with precision 73% using YOLOv5 in simulator experiments, while Horita et al. [35] applied YOLOv7 in laparoscopic colectomy, achieving average precision (AP)$_{50}$ of 57.4%. Hua et al. [13] reported precision of 84%, recall of 80%, and intersection over union (IoU) of 70% using Faster-RCNN.

Hong et al. [22] achieved an accuracy of 85% and an F1 score of 34%. Richter et al. [29] combined optical flow and CNNs for bleeding detection during robotic suction of blood and achieved IoU >50% in real-time bleeding scenarios. Sunakawa et al. [32] reported balanced Sørensen–Dice coefficient (Dice) of 77% and precision of 76%−79% during liver resections using DeepLabv3+. Tashiro et al. [31] tested DeepLabv3 overlays on laparoscopic liver resection videos and the results with a Dice score of 53% and an IoU of 42%.

Some researchers used classical computer vision for automatic bleeding detection. Jo et al. [26] reported sensitivity of 86% but variable specificity [33%–100%], while García-Martínez et al. [27] reported *in-vivo* accuracy of 78%. López et al. [28] evaluated classical CV techniques and CNNs for gauze tracking, achieving sensitivity >98% and specificity of 94% for the CNN model, which was superior.

#### Source tracking

Spatio-temporal hybrid models and dual-branch approaches integrating optical flow demonstrated bleeding source tracking, reporting Dice score of 65% and IoU of 79% [13, 37]. Robotic suction systems similarly achieved automated bleeding-source localization using CNNs and optical flow in real time [29] with an IoU of 79% - 80%. Daneshgar et al. [23] developed a system that can locate the source of bleeding during laparoscopic surgery using a local entropy filter.

#### Rate estimation and bleeding quantification

Gauze- and sponge-based approaches were explored in some studies, with gauze-image workflows reaching sensitivity of 96.5%, specificity of 98%, and mean absolute error (MAE) of 0.25 g, while smartphone-based models achieved $R^{2}$ of 93% and root mean squared error (RMSE) of 91% [25, 30]. Gauze-tracking quantification studies also confirmed sensitivity >98% and specificity of 94% [28].

In addition, robotic suction integrated with optical flow enabled real-time tracking of blood flow volumes alongside detection [29]. Other CNN-based detection systems, though not explicitly focused on rate estimation, suggested that their architectures could be adapted to support continuous bleeding quantification in surgical workflows [14, 35].

### Real-time feasibility

Evaluation of real-time feasibility was variably reported across the 21 included studies. Marullo et al. [14] reported successful processing of surgical videos at $\sim$21 frames per second (FPS) on a graphics processing unit (GPU), and Hua et al. [13] confirmed real-time detection and source tracking with Faster R-CNN in spatiotemporal pipelines. Horita et al. reported 30 FPS operation using YOLOv7 on laparoscopic colectomy videos [35], while Rus et al. reported response times under 1 s with YOLOv5 in simulation environments [24].

Sunakawa et al. [32] attained inference performance of 61.7 ms per frame using DeepLabv3+ in laparoscopic hepatectomy, supporting intraoperative deployment. Tashiro et al. [31] reported their system to be faster than 0.12 s for vascular recognition in laparoscopic liver resection. López et al. [28] measured 0.15 ms per frame for classical CV gauze tracking, but the CNN component required $\sim$4.6 s for processing.

Daneshgar et al. [23] reported latency of 0.12 ms for entropy-based segmentation using MATLAB in simulation. Jo et al. [26] achieved detection within 0.37 s per frame, also limited to MATLAB testing. García-Martínez et al. [27] operated at $\sim$24 FPS *in vivo* with OpenCV–Robot Operating System (ROS) pipelines.

Other studies did not explicitly quantify runtime or hardware constraints, often reporting real-time feasibility without detailed benchmarks. Notably, Hong et al. [22], and Rabbani et al. [38] validated their models in retrospective or simulation contexts but did not report frame rates and hardware performance.

## Critical analysis and synthesis

The 21 included studies demonstrate rapid progress in AI-driven bleeding monitoring, yet also expose recurrent limitations that constrain clinical translation.

### Risk of bias assessment

A primary source of bias identified across several early and prototype-based studies stems from the Analysis Domain (D4) [11, 12, 23]. These works frequently relied on limited sample sizes or failed to provide evidence of patient-level data splitting, which increases the likelihood of model overfitting and optimistic performance reporting. Furthermore, significant Participant Selection Bias (D1) was observed in studies utilizing non-clinical data sources; for instance, the use of animal-organ simulators [28] limits the generalizability of these findings to the complexities of real human laparoscopic surgery. However, a distinct shift toward higher methodological quality is evident in the most recent literature from 2024 and 2025. Studies such as those by Yoon et al. [25], Sunakawa et al. [32], Horita et al. [35], and Pei et al. [37] consistently achieved Low Risk ratings by employing multi-institutional human video databases and adhering to robust validation protocols, signaling a transition from experimental proof-of-concepts toward clinically transferable monitoring systems.

### Algorithmic approaches, performance, and cross-study comparison

Modern CNN and transformer pipelines achieved consistently high performance, with accuracy in the 90%–97% range. Real-time feasibility was reported in 12 of the studies, confirming technical potential. However, performance varied substantially by task and dataset. Early classical CV prototypes, [23, 26, 27, 39], performed more efficiently on CPUs but achieved lower accuracy and lacked adaptability. Benchmarking was further complicated by heterogeneous reporting of metrics, making cross-study comparisons difficult.

Across the included studies, divergence in reported performance and applicability appears to be driven primarily by differences in dataset size and quality, heterogeneous definitions of bleeding (frame-level, event-level, or pixel-level), domain shifts across procedures and institutions, and inconsistent reporting of evaluation metrics.

With respect to integration and deployment, a subset of studies demonstrated incorporation of bleeding-monitoring algorithms into surgical workflows, including robotic console overlays [33], autonomous suction systems [29], augmented-reality visualizations [24], vascular guidance overlays [31], and gauze-based blood loss quantification systems [25, 28, 30]. Nevertheless, the majority of proposed systems remained standalone prototypes or were evaluated solely on retrospective datasets without live operating room testing [11, 13, 22, 23, 26, 37, 38].

Most studies relied primarily on red–green–blue (RGB) laparoscopic video data, although some incorporated additional modalities such as gauze or sponge images for estimating blood loss [25, 28, 30], vascular overlays for intraoperative guidance [31, 36], and hybrid RGB plus optical-flow inputs [13, 29, 37]. Finally, while internal validation, expert comparison, and the release of public datasets were reported in some studies [13, 34, 35], external validation remains rare, with restricted data sharing and domain shifts continuing to limit generalizability [11, 25, 30, 38].

### Clinical validation and applicability

Several pipelines demonstrated direct intraoperative applicability, such as Horita et al.’s [35] nationwide colectomy dataset, Yoon et al.’s [25] gauze-based prospective human trial, and Richter et al.’s [29] autonomous robotic suction prototype. These provided strong evidence of real-time clinical relevance. Most studies remained retrospective, simulation-based, and single-center studies, with little evidence of prospective evaluation. Latency reporting was omitted in 42% of the studies, leaving practical deployment characteristics unclear.

It should be noted that no included study reported direct patient outcome measures, such as blood transfusion rates, operative mortality, or rates of conversion to open surgery. Clinical benefit therefore remains inferred from technical performance rather than demonstrated through prospective patient-level evidence.

### Data quality, datasets, and study limitations

Dataset size, diversity, and annotation strategies showed considerable variability across the 21 included studies. Most systems were developed on relatively small, single-institution datasets. For example, [12, 13, 31, 32] trained and tested models on laparoscopic videos from their own centers, with annotations performed by local experts. Checcucci et al. [33] developed the BLAIR system exclusively on robotic prostatectomy videos from a single hospital, while Bamba et al. [11] relied on just five colorectal surgery cases with 250 annotated bleeding objects. These approaches demonstrated strong within-dataset accuracy but lacked external validation, limiting transferability.

By contrast, only a few works leveraged broader or multi-institutional data. Acharya et al. [34] employed the Gynecologic Laparoscopy ENdometriosis DAtaset (GLENDA) of 400 gynecologic laparoscopies, a public benchmark spanning multiple centers, enabling stronger claims of robustness. Horita et al. [35] similarly reported bleeding detection from 27 colectomy cases across different Japanese institutions, supported by over 34,000 annotated images and consensus review by five surgeons, providing one of the most generalizable pipelines in this review.

Annotation practices also varied widely as shown in Table 1. Yoon et al. [25], López [28], and Abacı and Soyigazi [30] used gauze-based datasets with objective ground truth derived from blood weights, offering quantifiable measures for estimated blood loss (EBL). Pei et al. [37] annotated both bleeding regions and source points in the SurgBlood dataset, while Hua et al. [13] combined bounding boxes with optical flow maps. In contrast, earlier works [23, 26, 27, 39] relied on handcrafted thresholds, entropy filters, or tool-motion heuristics, with minimal labeling.

Purpose-built bleeding datasets such as SurgBlood [37], GLENDA [34], and the Japanese nationwide colectomy cohort [35], provided valuable benchmarks and reproducibility. Detailed annotations such as source-point coordinates and gauze weight–based ground truth strengthened method comparison and reproducibility. Still, dataset size and diversity were generally limited. Single-institution datasets dominated, and cross-institution validation was rare.

Several recurring study limitations were closely tied to dataset construction and annotation choices. Algorithmic performance frequently degraded under complex intraoperative conditions, including heavy bleeding, rapid camera motion, smoke, specular reflections, or poor illumination [24, 26, 27, 38]. Many approaches depended heavily on manual expert annotations (pixel-wise masks, bounding boxes, or precise event timing), which are labor-intensive and susceptible to inter-observer variability [11, 13, 25, 35]. Finally, rare but clinically critical hemorrhagic events were underrepresented in many datasets, restricting sensitivity to high-impact scenarios [32, 33, 35].

This synthesis is also subject to limitations of the review process. The included studies were heterogeneous in surgical specialty, data sources, outcome definitions, and evaluation protocols, which limited comparability and precluded quantitative meta-analysis. Screening and inclusion decisions were based on the reporting available in the manuscripts; incomplete methodological details (e.g., dataset composition, annotation procedures, latency and external validation) may therefore have led to misclassification and underestimation of deployment readiness. In addition, the search strategy may have missed relevant gray literature, non-English publications, and very recent conference or preprint work, and publication bias toward positive technical results may have inflated the apparent performance of proposed systems.

### Workflow integration, deployment barriers, and real-time utility

Marullo et al. [14] integrated a multi-task CNN into surgical video analysis with a demonstration in the operating room (OR). Hua et al. [13] confirmed real-time detection and source tracking in laparoscopic pipelines, while Horita et al. [35] applied YOLOv7 during colectomy procedures at 30 FPS. García-Martínez et al. [27] implemented OpenCV and ROS pipelines with both *in vivo* and *in vitro* testing. Checcucci et al. [33] embedded a bleeding alert overlay into the robotic console interface, providing intraoperative feedback to surgeons.

Several studies advanced toward clinical utility by addressing cumulative blood loss estimation. Yoon et al. [25] validated a gauze-image workflow against actual surgical sponges, reporting sensitivity of 95.6% and specificity of 98%. Abacı & Soygazi [30] applied smartphone-based machine learning to gauze images, achieving R$^{2}$ of 93% and RMSE of 91%. López et al. [28] conducted gauze-tracking quantification with sensitivity >98% and specificity of 94%. Richter et al. [29] integrated bleeding detection with a surgical robotic suction system.

The majority of the systems were assessed only in retrospective or simulated environments. Hong et al. [22] validated a fusion model on retrospective laparoscopic videos. Rus et al. [24] tested YOLOv5 in a simulator with strong recall but without live deployment. Rabbani et al. [38] employed a spatio-temporal memory model with domain adaptation using retrospective videos. Acharya et al. [34] trained EfficientNet-based models on general laparoscopy scenes using high-performance computing, but not in operative environments. Pei et al. [37] developed a dual-branch network for bleeding-source tracking, validated on annotated datasets rather than live integration.

Several early studies remained proof-of-concept. Daneshgar et al. [23, 39] evaluated entropy-based segmentation and predictive models in MATLAB. Jo et al. [26] explored color segmentation with Otsu thresholding. Tashiro et al. [31] introduced a DeepLabv3 overlay system for vascular recognition in liver resection. Sunakawa et al. [32] improved on this using DeepLabv3+, achieving Dice score of 77% in liver resections. Li et al. [36] proposed a trocar-mounted classification device for vascular vs avascular tissue, though validation was limited to simulation.

Fifty-two percent of the studies reported real-time applicability, particularly those based on GPU-accelerated CNNs and those based on classical computer vision pipelines. Some achieved meaningful workflow integration: the BLAIR system [33] provided console alerts, and Richter et al. [29] enabled closed-loop suction control. Gauze-based systems offered objective quantification, a potential asset for perioperative management. Most pipelines remained GPU-intensive, with limited suitability for embedded OR hardware. Structured usability or ergonomic testing with surgeons was rarely performed, which leaves integration into surgical workflows under-explored.

Although bleeding detection and quantification models demonstrated strong technical performance, multiple barriers limit their translation to safe and routine OR use. Compute and latency constraints were frequently reported. CNN- and transformer-based models reported in the studies, [11–13, 22, 34, 35, 37], relied on GPU acceleration to achieve real-time, with frame rates ranging from 20–48 fps. While effective, such dependence raises concerns for deployment on embedded OR hardware. Rus et al. [24] and Richter et al. [29] confirmed real-time feasibility with YOLOv5 and CNN–optical flow respectively, though both remained GPU-intensive. In contrast, classical computer vision systems reported by [23, 26, 27] and [39] ran efficiently on CPUs, achieving sub-second responses, but at the cost of lower accuracy and robustness.

Interoperability and workflow integration were rarely addressed beyond prototypes. Checcucci et al.’s [33] BLAIR system represented a notable example, embedding bleeding alerts directly into a robotic prostatectomy console, while Richter et al. [29] integrated autonomous robotic suction for blood clearance. Rus et al. [24] combined YOLOv5 with HoloLens for augmented-reality overlays in robotic single-incision surgery. Tashiro et al. [31] and Sunakawa et al. [32] provided color-coded overlays during liver resections, supporting intraoperative awareness. However, other systems remained stand-alone pipelines. The gauze-based workflows [25, 28, 30] also remained disconnected from perioperative systems.

User interfaces and ergonomics received little evaluation. López et al. [28] aligned gauze tracking with alarms and video markers for surgeon support, while Tashiro et al. [31] and Sunakawa et al. [32] produced intuitive overlays during liver resection [28, 31, 32]. A robotic suction prototype by [29] was reported to reduce cognitive and manual burden by automating hemostasis. Checcucci et al. [33] implemented a PyQT-based graphical user interface (GUI) for real-time alerts, and Acharya et al. [34] developed a GUI platform to visualize EfficientNet predictions [34]. By contrast, other works, [13, 22, 37, 38] lacked surgeon-facing interfaces, limiting ergonomic value.

Explainability and trust remained major challenges. Most CNN pipelines, [11, 13, 14, 22], operated as black boxes without post-hoc interpretation. Some strategies showed promise. Daneshgar et al. [23] and Daneshgar et al. [39] developed systems with outputs of entropy maps and tool-motion entropy predictions providing interpretable outputs. Rus et al. [24] developed a system with augmented-reality overlays and Richter et al. [29] developed a system showing blood-flow trajectories. Tashiro et al. [31] and Sunakawa et al. [32] developed systems that had color-coded segmentation maps, which aid interpretability [31, 32], Acharya et al. [34] incorporated explainable AI visualizations and Checcucci et al. [33] benchmarked AI alerts against urologists, reinforcing clinical trust. Li et al. [36] developed a probe-based approach that leveraged interpretable physiological metrics to justify outputs, while Rabbani et al. [38] employed domain adaptation to reduce dataset bias and improve surgeon confidence.

Although bleeding detection and quantification models demonstrated strong technical performance, multiple barriers limit their translation to safe and routine OR use. Compute and latency constraints were frequently reported. CNN- and transformer-based models reported in the studies, [11–13, 22, 34, 35, 37], relied on GPU acceleration to achieve real-time, with frame rates ranging from 20–48 fps. While effective, such dependence raises concerns for deployment on embedded OR hardware. Rus et al. [24] and Richter et al. [29] confirmed real-time feasibility with YOLOv5 and CNN + optical flow respectively, though both remained GPU-intensive. In contrast, classical computer vision systems reported by [23, 26, 27] and [39] ran efficiently on CPUs, achieving sub-second responses, but at the cost of lower accuracy and robustness.

Interoperability and workflow integration were rarely addressed beyond prototypes. Checcucci et al.’s [33] BLAIR system represented a notable example, embedding bleeding alerts directly into a robotic prostatectomy console, while Richter et al. [29] integrated autonomous robotic suction for blood clearance. Rus et al. [24] combined YOLOv5 with HoloLens for augmented-reality overlays in robotic single-incision surgery. Tashiro et al. [31] and Sunakawa et al. [32] provided color-coded overlays during liver resections, supporting intraoperative awareness. However, other systems remained stand-alone pipelines. The gauze-based workflows [25, 28, 30] also remained disconnected from perioperative systems.

User interfaces and ergonomics received little evaluation. López et al. [28] aligned gauze tracking with alarms and video markers for surgeon support, while Tashiro et al [31] and Sunakawa et al. [32] produced intuitive overlays during liver resection [28, 31, 32]. A robotic suction prototype by [29] was reported to reduce cognitive and manual burden by automating hemostasis. Checcucci et al. [33] implemented a PyQT-based graphical user interface (GUI) for real-time alerts, and Acharya et al. [34] developed a GUI platform to visualize EfficientNet predictions [34]. By contrast, other works, [13, 22, 37, 38] lacked surgeon-facing interfaces, limiting ergonomic value.

Explainability and trust remained major challenges. Most CNN pipelines, [11, 13, 14, 22], operated as black boxes without post-hoc interpretation. Some strategies showed promise. Daneshgar et al. [23] and Daneshgar et al. [39] developed systems with outputs of entropy maps and tool-motion entropy predictions providing interpretable outputs. Rus et al. [24] developed a system with AR overlays and Richter et al. [29] developed a system showing blood-flow trajectories. Tashiro et al. [31] and Sunakawa et al. [32] developed systems that had color-coded segmentation maps, which aid interpretability [31, 32], Acharya et al. [34] incorporated explainable AI visualizations and Checcucci et al. [33] benchmarked AI alerts against urologists, reinforcing clinical trust. Li et al. [36] developed a probe-based approach that leveraged interpretable physiological metrics to justify outputs, while Rabbani et al. [38] employed domain adaptation to reduce dataset bias and improve surgeon confidence.

### Scope beyond bleeding detection

Li et al.’s [36] smart probe enabled vascular recognition, while gauze- and sponge-based systems provided continuous EBL estimation. Such innovations highlight the potential of AI-based bleeding monitoring to contribute to broader perioperative safety frameworks. Coverage across diverse procedures and bleeding phenotypes was uneven, and adaptability to variable OR conditions (e.g., lighting, camera motion, tool occlusion) remains underexplored. Multimodal sensing approaches are still in their infancy.

### Gaps and future research directions

Although considerable progress has been achieved across the 21 included studies, several gaps remain that shape future research directions. A first recurring gap concerns the reliance on RGB video as the dominant data modality. While effective in many contexts, this dependence makes models vulnerable to challenges such as smoke produced from cutting tissue of stopping bleeding using electrosurgical tools, poor illumination and rapid camera motion. Future work should explore multimodal strategies that combine RGB imaging with other imaging modalities such as infrared imaging, to improve robustness.

Another limitation lies in the scarcity of large, diverse, and richly annotated datasets. Most studies were based on single-center data, often with inconsistent labels. Building multi-institutional, standardized datasets with detailed pixel- and event-level annotations is therefore a priority. Addressing the ethical and privacy barriers to data sharing will require anonymization pipelines, federated learning (FL), frameworks [40], and strong governance models. FL is a machine learning approach that allows multiple devices and institutions to collaboratively train a shared model without directly sharing their raw data. Instead of sending data to a central server, each participant trains the model locally on its own data and then sends only the model updates to a central coordinator. The coordinator aggregates these updates to improve the global model, which is then redistributed back to the clients for further local training [40].

A persistent gap relates to ethical, legal, and privacy issues surrounding the use of surgical video data. Strict patient confidentiality requirements and regulatory frameworks such as the Protection of Personal Information Act (POPIA) in South Africa [41], the Health Insurance Portability and Accountability Act (HIPAA) in the United States [42], and the General Data Protection Regulation (GDPR) in the European Union create significant obstacles to data sharing and multi-center collaborations [43]. In South Africa, POPIA has been enforceable since 2021 and governs how personal and health information is collected, processed, and stored [44]. In the United States, HIPAA regulates the use and disclosure of protected health information across healthcare institutions. Similarly, the GDPR, in effect across the European Union provides strict standards for handling personal data and international data transfer. Many institutions remain hesitant to release annotated surgical datasets due to fear of medico-legal risk. Without solutions for secure anonymization, consent management, and cross-border governance, large-scale, diverse datasets will remain difficult to assemble. Addressing these barriers through FL approaches, privacy-preserving AI, and robust ethical frameworks will be essential to unlock wider validation and global collaboration.

Clinical validation remains limited. While retrospective evaluations and simulation-based prototypes are common, very few systems have been tested prospectively in real-world ORs. Future research must prioritize prospective, multi-center clinical trials that explicitly report latency, usability, and safety outcomes. Closely linked to this is the challenge of integration within surgical workflows. Many current pipelines require high-end GPU hardware or offline processing, limiting practical deployment. There is a clear need for lightweight, edge-optimized models capable of sustaining real-time inference, combined with user-centered interfaces that deliver intuitive overlays and alerts. Bleeding monitoring systems have largely been developed in isolation, with limited integration into broader digital surgery ecosystems such as telementoring applications or robotic platforms. Embedding bleeding detection and quantification modules into telementoring systems could enhance remote surgical training and support by providing real-time feedback to both mentor and trainee. Similarly, integration into robotic platforms offers opportunities for closed-loop assistance, such as automated suction or vessel recognition overlays during minimally invasive procedures. Future work should therefore move toward unified platforms where bleeding monitoring is not a standalone function, but part of a wider suite of intraoperative decision-support tools.

Another area requiring attention is AI-explainability. Most systems provide little interpretability beyond binary outputs, which restricts their usefulness in decision support. Incorporating attention maps, reliability indicators, and case-based examples tailored to intraoperative contexts would strengthen transparency and user confidence. Furthermore, the majority of bleeding-monitoring systems remain siloed, developed as standalone solutions. Future directions should aim to embed bleeding detection within broader surgical AI platforms that integrate intraoperative phase recognition, skills assessment and risk prediction, supporting more holistic intraoperative decision-making.

Methodological gaps also persist. Many studies rely on computationally heavy architectures that are difficult to sustain at surgical frame rates without dedicated GPUs. Research into neural network architectures, temporal sparsity, and streaming-friendly processing could reduce latency and improve scalability. Finally, there is little standardization in evaluation protocols. Task definitions vary widely; whether bleeding is labeled at frame, event, or pixel level, and metrics differ across studies. Establishing shared benchmarks, metric suites, and reporting standards will be critical for fair comparison, reproducibility, and regulatory acceptance.

## Conclusion

This systematic review synthesized evidence from 21 included studies from 332 records obtained through database and grey literature search investigating artificial intelligence for intraoperative bleeding monitoring during conventional and robotic-assisted laparoscopic surgery. Across diverse approaches ranging from early computer vision methods to modern CNN and transformer-based architectures, most systems demonstrated high detection accuracy in predominantly single-centre, retrospective, or simulation-based settings and promising real-time feasibility under controlled experimental conditions. Several works also extended beyond detection to address bleeding source localization, blood loss quantification, and workflow integration, highlighting the expanding scope of AI applications in surgical safety that remains potential, though largely unvalidated in prospective clinical settings.

Despite this progress, significant challenges remain. Many studies relied on limited, single-center datasets, and retrospective and simulated evaluations, which constrain generalizability. Latency reporting was inconsistent, and practical deployment often depended on GPU resources unavailable in many operating environments. Ethical and privacy concerns further limit large-scale dataset creation and sharing, while inconsistent definitions of bleeding and heterogeneous evaluation metrics hinder reproducibility and cross-study comparison. Moreover, only a small number of systems demonstrated genuine integration into real-time OR workflows, with most remaining proof-of-concept prototypes.

Future research could therefore prioritize the development of large, diverse, and standardized datasets, accompanied by transparent benchmarks and reporting guidelines. Prospective, multi-center clinical trials are required to validate performance in real-world settings, alongside innovations in lightweight, edge-optimized models suitable for OR deployment. Integration into broader surgical AI ecosystems, including telementoring platforms and robotic systems, will be critical to achieving holistic intraoperative decision support. Addressing AI-explainability, ethical, and governance concerns will also be central to earning surgeon trust and ensuring safe adoption.

In summary, AI-driven bleeding monitoring shows potential, though largely unvalidated in prospective clinical settings, to enhance surgical safety, situational awareness, and training. However, realizing its clinical impact will require a shift from technical feasibility demonstrations toward robust, standardized, and ethically grounded systems that are seamlessly embedded into surgical workflows and validated across diverse patient populations and institutions.

## Supplementary Information

Below is the link to the electronic supplementary material.

Supplementary file 1

## Data Availability

No datasets were generated or analysed during the current study.

## Appendix: A data extraction form

See Table 2.

**Table 2:** Data extraction form (adapted from the cochrane handbook)

Field	What to record (guidance)
Study ID	First author surname and year (e.g., “Surname YYYY”)
Study title	Full title of the article exactly as published
Authors	All listed authors in order of appearance
Year of publication	Year the article was published
Country	Country or region where the study was conducted
Study design	Study type (e.g., retrospective, prospective, RCT, technical evaluation)
Surgical setting	Context of data capture (clinical OR, simulation lab, dataset archive)
Laparoscopic procedure type	Specific laparoscopic procedure(s) included
Artificial intelligence technique	Model architecture and approach (e.g., CNN, U-Net, Transformer)
Framework/tools used	Programming frameworks, toolkits, and versions (e.g., PyTorch, TensorFlow)
Input data type	Type of input data (e.g., video, still images, modalities) used for model development
Resolution	Resolution of input data (e.g., 640x480, HD)
Annotation/ground truth source	Who provided labels (e.g., surgeons), labeling method, consensus, and reliability checks
AI task addressed	Purpose (detection, prediction, tracking, quantification, segmentation)
Performance metrics reported	All reported metrics (accuracy, sensitivity, specificity, F1 score, latency, etc.)
Validation approach	Internal validation (train/test split, k-fold) and external validation (multi-site, other dataset)
Comparator	Baseline method, traditional approach, clinician assessment, or none
Clinical relevance reported	Any comments on real-time feasibility, OR applications, or workflow integration
Inclusion criteria met	Indicate whether this study meets inclusion criteria for the review (yes/no)
Reason for exclusion (if applicable)	If excluded, specify why (map to your exclusion criteria)
Final decision	Include or exclude, and for which synthesis (qualitative/quantitative)
Risk of bias summary	Judgement using the chosen tool (e.g., ROBINS-I, QUADAS-2), with brief rationale
Reviewer comments	Notes from the reviewer, clarifications, or concerns

Template adapted from the Cochrane *Handbook for Systematic Reviews of Interventions* [20], tailored to AI in laparoscopic surgery
