# Supplementary material for: AI-based automated bleeding monitoring in conventional and robot-assisted laparoscopic surgery: a systematic review
Source: J Robot Surg. 2026 May 23;20(1):522. doi: 10.1007/s11701-026-03469-4 (PMC13197375; doi:10.1007/s11701-026-03469-4)
Supplement: Supplementary file 1 [file 11701_2026_3469_MOESM1_ESM.pdf]

PRISMA 2020 Checklist

Section and Topic	Item #	Checklist item	Location where item is reported
**TITLE**			
Title	1	Identify the report as a systematic review.	Title page. Title reads: 'AI-Based Automated Bleeding Monitoring in Conventional and Robot-Assisted Laparoscopic Surgery: A Systematic Review.'
**ABSTRACT**			
Abstract	2	See the PRISMA 2020 for Abstracts checklist.	pp. 1-2. Abstract reports background, PRISMA-compliant methods, 21 included studies, qualified performance claims, and conclusions.
**INTRODUCTION**			
Rationale	3	Describe the rationale for the review in the context of existing knowledge.	pp. 2-3. Rationale addresses gaps in static AI methods and inadequately addressed real-time bleeding detection in conventional and robotic-assisted laparoscopic surgery.
Objectives	4	Provide an explicit statement of the objective(s) or question(s) the review addresses.	p. 3, Section 2.2. Three predefined research questions covering AI systems, performance metrics, and real-time feasibility vs. classical CV methods.
**METHODS**			
Eligibility criteria	5	Specify the inclusion and exclusion criteria for the review and how studies were grouped for the syntheses.	p. 4, Section 3.1. PICO framework; population defined as patients, animal models, and simulation-based datasets. Robotic-assisted procedures explicitly included. Primary and secondary outcomes stated.
Information sources	6	Specify all databases, registers, websites, organisations, reference lists and other sources searched or consulted to identify studies. Specify the date when each source was last searched or consulted.	p. 5, Section 3.2. PubMed, Scopus, Web of Science, IEEE Xplore, Embase, EBSCOHost, ProQuest, Google Scholar, arXiv, MedRxiv. Search concluded 22 July 2025.
Search strategy	7	Present the full search strategies for all databases, registers and websites, including any filters and limits used.	p. 5, Section 3.2. Full PubMed Boolean string provided; equivalent Boolean-adapted strategies applied across all databases.
Selection process	8	Specify the methods used to decide whether a study met the inclusion criteria of the review, including how many reviewers screened each record and each report retrieved, whether they worked independently, and if applicable, details of automation tools used in the process.	pp. 5-6, Section 3.3. Two-stage screening in Rayyan; Reviewers PM and AB independently screened titles/abstracts; Reviewer BM resolved disagreements.
Data collection process	9	Specify the methods used to collect data from reports, including how many reviewers collected data from each report, whether they worked independently, any processes for obtaining or confirming data from study	p. 5, Section 3.3; Appendix A, pp. 19-20. Cochrane-adapted extraction form used;

Section and Topic	Item #	Checklist item	Location where item is reported
		investigators, and if applicable, details of automation tools used in the process.	Reviewer PM extracted data, Reviewer AB independently verified decisions.
Data items	10a	List and define all outcomes for which data were sought. Specify whether all results that were compatible with each outcome domain in each study were sought (e.g. for all measures, time points, analyses), and if not, the methods used to decide which results to collect.	p. 4, Section 3.1. Primary outcome: AI model detection performance. Secondary outcomes: latency and real-time feasibility, workflow integration feasibility, and dataset characteristics.
	10b	List and define all other variables for which data were sought (e.g. participant and intervention characteristics, funding sources). Describe any assumptions made about any missing or unclear information.	Appendix A, pp. 19-20. Variables include study design, surgical setting, AI technique, input data type, annotation source, validation approach, and comparator.
Study risk of bias assessment	11	Specify the methods used to assess risk of bias in the included studies, including details of the tool(s) used, how many reviewers assessed each study and whether they worked independently, and if applicable, details of automation tools used in the process.	p. 5, Section 3.3; Figure 2, p. 8. PROBAST framework applied independently by Reviewers PM and AB across four domains: Participants, Predictors, Outcome, and Analysis.
Effect measures	12	Specify for each outcome the effect measure(s) (e.g. risk ratio, mean difference) used in the synthesis or presentation of results.	Table 1, p. 9; Section 4, pp. 6-11. Accuracy, precision, recall, F1, Dice, IoU, sensitivity, specificity, RMSE, R2, MAE, AP50, and FPS reported per study as applicable.
Synthesis methods	13a	Describe the processes used to decide which studies were eligible for each synthesis (e.g. tabulating the study intervention characteristics and comparing against the planned groups for each synthesis (item #5)).	p. 4, Section 3.1; p. 6, Section 4. Studies grouped narratively by AI task: prediction, detection, source tracking, and rate estimation/quantification.
	13b	Describe any methods required to prepare the data for presentation or synthesis, such as handling of missing summary statistics, or data conversions.	Table 1, p. 9. Missing latency or metrics noted as NR (Not Reported); no statistical conversion performed.
	13c	Describe any methods used to tabulate or visually display results of individual studies and syntheses.	Table 1, p. 9; Figure 1, p. 7; Figure 2, p. 8. Descriptive summary table, PRISMA flow diagram, and PROBAST traffic light plot used.
	13d	Describe any methods used to synthesize results and provide a rationale for the choice(s). If meta-analysis was performed, describe the model(s), method(s) to identify the presence and extent of statistical heterogeneity, and software package(s) used.	p. 6, Section 4. Narrative synthesis used; meta-analysis not feasible due to heterogeneous outcome definitions — bleeding defined at frame, pixel, and event level — and inconsistent metric reporting.
	13e	Describe any methods used to explore possible causes of heterogeneity among study results (e.g. subgroup analysis, meta-regression).	p. 12, Section 5.2. Heterogeneity explored narratively: dataset size, bleeding definitions, domain shifts across procedures and institutions, and

PRISMA

PRISMA 2020 Checklist

Section and Topic	Item #	Checklist item	Location where item is reported
			inconsistent metric reporting.
	13f	Describe any sensitivity analyses conducted to assess robustness of the synthesized results.	N/A. No quantitative meta-analysis conducted; study-level risk of bias assessed via PROBAST (Section 5.1, p. 11).
Reporting bias assessment	14	Describe any methods used to assess risk of bias due to missing results in a synthesis (arising from reporting biases).	pp. 12-13, Sections 5.2-5.3. Latency unreported in 42% of studies noted; retrospective and simulated evaluations identified as sources of reporting bias.
Certainty assessment	15	Describe any methods used to assess certainty (or confidence) in the body of evidence for an outcome.	p. 5, Section 3.3; Figure 2, p. 8. PROBAST used for methodological quality assessment; overall certainty discussed narratively in Sections 5.1-5.3.
**RESULTS**			
Study selection	16a	Describe the results of the search and selection process, from the number of records identified in the search to the number of studies included in the review, ideally using a flow diagram.	pp. 5-6, Section 3.3; Figure 1, p. 7. 332 records identified; 117 duplicates removed; 215 screened by title; 61 by abstract; 37 full texts reviewed; 21 included.
	16b	Cite studies that might appear to meet the inclusion criteria, but which were excluded, and explain why they were excluded.	p. 6, Section 3.3. Excluded full texts documented in Rayyan; reasons: general surgery focus, endoscopy focus, or non-AI bleeding monitoring methods.
Study characteristics	17	Cite each included study and present its characteristics.	Table 1, p. 9. All 21 included studies cited with detection accuracy, response time, integration feasibility, and algorithmic complexity.
Risk of bias in studies	18	Present assessments of risk of bias for each included study.	Figure 2, p. 8. PROBAST traffic light plot for all 21 studies across four domains with Low/Moderate/High overall risk ratings.
Results of individual studies	19	For all outcomes, present, for each study: (a) summary statistics for each group (where appropriate) and (b) an effect estimate and its precision (e.g. confidence/credible interval), ideally using structured tables or plots.	pp. 8-11; Table 1, p. 9. Per-study performance metrics, response times, and integration feasibility reported for all 21 included studies.
Results of syntheses	20a	For each synthesis, briefly summarise the characteristics and risk of bias among contributing studies.	pp. 11-12, Sections 5.1-5.2. Characteristics and risk of bias summarised by AI task group and publication period.
	20b	Present results of all statistical syntheses conducted. If meta-analysis was done, present for each the summary estimate and its precision (e.g. confidence/credible interval) and measures of statistical heterogeneity. If comparing groups, describe the direction of the effect.	pp. 8-11, Section 4. Narrative results presented by task: prediction, detection, source tracking, quantification, and real-time feasibility.

PRISMA

PRISMA 2020 Checklist

Section and Topic	Item #	Checklist item	Location where item is reported
	20c	Present results of all investigations of possible causes of heterogeneity among study results.	p. 12, Section 5.2. Heterogeneity sources: dataset size and quality, bleeding definitions, domain shifts, and inconsistent metric reporting.
	20d	Present results of all sensitivity analyses conducted to assess the robustness of the synthesized results.	N/A. No quantitative meta-analysis or sensitivity analysis was conducted.
Reporting biases	21	Present assessments of risk of bias due to missing results (arising from reporting biases) for each synthesis assessed.	pp. 12-13, Sections 5.2-5.3. Latency omitted in 42% of studies; retrospective and simulated validation prevalent across included studies.
Certainty of evidence	22	Present assessments of certainty (or confidence) in the body of evidence for each outcome assessed.	pp. 11, 18, Sections 5.1 and 6. Certainty assessed as promising but limited by single-centre designs, retrospective validation, and inconsistent metric reporting.
**DISCUSSION**			
Discussion	23a	Provide a general interpretation of the results in the context of other evidence.	pp. 11-17, Section 5; p. 18, Section 6. Results interpreted against technical maturity, clinical translation readiness, and the surgical AI literature.
	23b	Discuss any limitations of the evidence included in the review.	pp. 13-14, Section 5.4. Limitations: small/single-centre datasets, retrospective analyses, heterogeneous metrics, absent latency reporting, and annotation bias.
	23c	Discuss any limitations of the review processes used.	p. 13, Section 5.4. Inclusion limited to English-language peer-reviewed publications; grey literature supplemented via Google Scholar, arXiv, and MedRxiv.
	23d	Discuss implications of the results for practice, policy, and future research.	pp. 15-17, Sections 5.5-5.7. Implications for OR deployment, federated learning, POPIA/HIPAA/GDPR compliance, telementoring, and robotic platform integration.
**OTHER INFORMATION**			
Registration and protocol	24a	Provide registration information for the review, including register name and registration number, or state that the review was not registered.	p. 4, Section 3. Protocol registered on the Open Science Framework (OSF); registration number NCEF8 (https://doi.org/10.17605/OSF.IO/NCEF8) [Ref. 16].
	24b	Indicate where the review protocol can be accessed, or state that a protocol was not prepared.	p. 4, Section 3. OSF registration accessible at https://doi.org/10.17605/OSF.IO/NCEF8

PRISMA 2020 Checklist

Section and Topic	Item #	Checklist item	Location where item is reported
	24c	Describe and explain any amendments to information provided at registration or in the protocol.	The Title has been changed to match the title changes recommended by the reviewers of this systematic review.
Support	25	Describe sources of financial or non-financial support for the review, and the role of the funders or sponsors in the review.	p. 19, Section 7. EU Grant DCI-PANAF/2020/420-028 via ARISE/AAS; Telkom South Africa CoE; Google Fellowship Programme; Carnegie Corporation of New York (DEAL Postdoctoral Fellowship).
Competing interests	26	Declare any competing interests of review authors.	p. 19, Section 7. Declared: 'No conflicts of interest to declare.'
Availability of data, code and other materials	27	Report which of the following are publicly available and where they can be found: template data collection forms; data extracted from included studies; data used for all analyses; analytic code; any other materials used in the review.	Appendix A, pp. 19-20. Data extraction form template provided. Publicly available datasets referenced: GLENDA [Ref. 34] and SurgBlood [Ref. 37]. Extracted data not explicitly deposited.

From: Page MJ, McKenzie JE, Bossuyt PM, Boutron I, Hoffmann TC, Mulrow CD, et al. The PRISMA 2020 statement: an updated guideline for reporting systematic reviews. BMJ 2021;372:n71. doi: 10.1136/bmj.n71. This work is licensed under CC BY 4.0. To view a copy of this license, visit https://creativecommons.org/licenses/by/4.0/
